# Norepinephrine Regulation of Ventromedial Hypothalamic Nucleus Metabolic-Sensory Neuron 5′-AMP-Activated Protein Kinase Activity: Impact of Estradiol

**DOI:** 10.3390/ijms21062013

**Published:** 2020-03-16

**Authors:** A. S. M. Hasan Mahmood, Md. Main Uddin, Mostafa M. H. Ibrahim, Karen P. Briski

**Affiliations:** School of Basic Pharmaceutical and Toxicological Sciences, College of Pharmacy, University of Louisiana Monroe, Monroe, LA 71201, USA; mahmooas@warhawks.ulm.edu (A.S.M.H.M.); uddnmm@warhawks.ulm.edu (M.M.U.); ibrahimm@warhawks.ulm.edu (M.M.H.I.)

**Keywords:** ventromedial hypothalamic nucleus, norepinephrine, estrogen receptor, nitric oxide synthase, glutamate decarboxylase, laser-catapult microdissection

## Abstract

The mediobasal hypothalamus (MBH) shapes the neural regulation of glucostasis by 5′-AMP-activated protein kinase (AMPK)-dependent mechanisms. Yet, the neurochemical identity and neuroanatomical distribution of MBH neurons that express glucoprivic-sensitive AMPK remain unclear. The neurotransmitters γ-aminobutyric acid (GABA) and nitric oxide (NO) act within the MBH to correspondingly inhibit or stimulate glucose counter-regulation. The current review highlights recent findings that GABA and NO, neurons located in the ventromedial hypothalamic nucleus (VMN), a distinct important element of the MBH, are direct targets of noradrenergic regulatory signaling, and thereby, likely operate under the control of hindbrain metabolic-sensory neurons. The ovarian hormone estradiol acts within the VMN to govern energy homeostasis. Discussed here is current evidence that estradiol regulates GABA and NO nerve cell receptivity to norepinephrine and moreover, controls the noradrenergic regulation of AMPK activity in each cell type. Future gains in insight on mechanisms underpinning estradiol’s impact on neurotransmitter communication between the hindbrain and hypothalamic AMPKergic neurons are expected to disclose viable new molecular targets for the therapeutic simulation of hormonal enhancement of neuro-metabolic stability during circumstances of diminished endogenous estrogen secretion or glucose dysregulation.

**Introduction**: Insulin-induced hypoglycemia (IIH) is a relentless, worrisome complication of the meticulous therapeutic management of type I and long-term type II diabetes mellitus patients, and is a major impediment to optimal glycemic control. IIH poses a risk of neurological dysfunction and injury as decrements in glucose supply, the primary fuel source to the brain, impede vital high-energy-demand nerve cell functions, including the maintenance of transmembrane electrolyte gradients. Hypoglycemia-associated neuro-glucopenia triggers counteractive, autonomic, neuroendocrine, and behavioral responses that heighten circulating glucose levels. The ventromedial hypothalamic nucleus (VMN), a distinct unique component of the mediobasal hypothalamus (MBH) and a key element of the brain’s gluco-regulatory network, processes nutrient, endocrine, and neurochemical cues on a metabolic status to control glucose counter-regulation [1,2]. The detection of neuro-energetic sequelae of hypoglycemia in the MBH is vital for optimum counter-regulatory endocrine and gluconeogenic outflow [3,4]. MBH metabolic-sensory neurons produce a dynamic cellular energy readout through the augmentation or diminution of the synaptic firing rate as ambient glucose levels fall [5,6,7]. Neurochemical effectors of MBH energy imbalance include the amino acid γ-aminobutyric acid (GABA), which inhibits glucagon and adrenomedullary catecholamine secretory responses to IIH [8], and the gluco-stimulatory gaseous transmitter nitric oxide (NO), which intensifies counter-regulatory hormone secretion [9,10].

**Role of the MBH ultra-sensitive energy gauge 5′-AMP-activated protein kinase (AMPK) in glucose homeostasis**. AMPK operates within the MBH to optimize hypoglycemic patterns of NO transmission [11] and counter-regulatory hormone release [12,13]. MBH AMPK is a common focal substrate for an array of nutrient and endocrine indicators of energy paucity (ghrelin, corticosterone, thyroxine, adiponectin) or excess (glucose, leptin, insulin) [14], where these diverse cues are integrated to shape systemic energy homeostasis through the regulation of food intake, glucose production, energy expenditure, and body weight [15]. Substrate fuel screening in the caudal dorsomedial hindbrain has a critical impact on neural gluco-regulation, as the local diminution of the oxidizable glycolytic end-product l-lactate causes hyperglycemia, while an exogenous lactate infusion to this site aggravates hypoglycemia [16]. Recent studies documented the hindbrain AMPK influence of MBH sensor energy activity [17,18]. Hindbrain AMPK inhibition attenuates the hypoglycemic up-regulation of VMN AMPK activity and the local expression of the NO marker protein neuronal nitric oxide synthase (nNOS) [19]. 

**Hindbrain control of VMN gluco-regulation**. Communication between hindbrain and VMN sensors is presumably mediated, in part, by the catecholamine (CA) transmitter norepinephrine (NE), as the administration of the AMPK inhibitor compound C (Cc) into the hindbrain prevents hypoglycemic patterns of VMN NE activity [19], and hindbrain CA nerve cell lesions avert the hindbrain lactoprivic augmentation of MBH AMPK activity, circulating glucose and glucagon levels, and food intake [20]. Located in the caudal dorsomedial hindbrain, A2 noradrenergic neurons express hypoglycemia-sensitive metabolic-sensory biomarkers, e.g., glucokinase, K_ATP_, and AMPK [21,22]. These cells likely detect the energetic sequelae of hypoglycemia as AMPK is activated in a lactate-reversible manner in this (but not other) hindbrain’s catecholamine cell groups, alongside lactoprivic-dependent increases in hypothalamic NE activity [23]. The direct delivery of NE to the VMN alters metabolic transmitter marker proteins, e.g., nNOS and glutamate decarboxylase_65/67_ (GAD) expression [24]. Interestingly, Cc pretreatment of the VMN normalizes local patterns of nNOS expression in NE-treated animals. Due to the complex neurochemical heterogeneity of the VMN, studies focusing on individual neurotransmitter cell populations require high-neuroanatomical resolution dissection tools for the procurement of pure nerve cell samples. Using combinatory immunocytochemistry/laser-catapult microdissection for the discriminative harvesting of VMN NO and GABA neurons, our studies revealed that each population is likely a direct target for noradrenergic input as these nerve cells express alpha_1_- (α_1_-), alpha_2_- (α_2_-), and beta_1_- (β_1_-) adrenoreceptor (AR) proteins [24]. 

**Estrogen control of gluco-regulation**. The ovarian steroid hormone estradiol controls metabolic homeostasis through the regulation of the procurement, ingestion, metabolism, partitioning, storage, and expenditure of energy fuels [25]. In the female rat, estradiol regulates hypoglycemia-associated neuronal transcriptional activation in key hypothalamic metabolic loci [26], as well as the hyperphagic, hyperglucagonemic, and hypercorticosteronemic responses to hypoglycemia in ovariectomized (OVX) animals [27]. In that sense, estradiol modulates the hindbrain AMPK regulation of circulating glucose and counter-regulatory hormone profiles and effects on NE signaling, neuronal transcriptional activation, AMPK activation, and metabolic effector transmitter expression in the hypothalamus, including the VMN [17,18,28]. Observations that A2 neurons express estrogen receptor-alpha (ERα) and -beta (ERβ) proteins [28] infer that estradiol may act directly on these metabolo-sensory cells to control local metabolic screening and functional interaction with neuroanatomically distant energy sensors. The VMN is also a plausible site of the estrogenic control of glucostasis as the local administration of estradiol alters insulin-induced hypoglycemia in OVX female rats [29]. Documented effects of the intracerebroventricular (*icv*) delivery of ERα antagonist 1,3-*Bis*(4-hydroxyphenyl)-4-methyl-5-[4-(2-piperidinylethoxy)phenol]-1*H*-pyrazole dihydrochloride (MPP) or ERβ antagonist 4-[2-phenyl-5,7-*bis*(trifluoromethyl)pyrazolo[1,5-*a*]pyrimidin-3-yl]phenol (PHTPP) implicate these estrogen receptors (ERs) in forebrain mechanisms that govern VMN nNOS and GAD_65/67_ protein responses to hypoglycemia [30].

**Estrogen regulation of noradrenergic input to VMN gluco-regulatory neurons**. VMN NO and GABA neurons are presumptive objects of NE and estradiol action as each cell type expresses ERα, ERβ, and the transmembrane G protein-coupled estrogen receptor GPER/GPR30 proteins [31]. ERs regulate the hypoglycemic patterns of AR protein expression in these neurons in a cell type-specific manner. Hypoglycemia elicits divergent adjustments in α_1_-AR protein expression in nitrergic (up-regulated) versus GABAergic (down-regulated) neurons, responses that are respectively mediated by ERβ alone versus ERα and ERβ signaling. Moreover, NO and GABA neuron β_1_-AR profiles are decreased during hypoglycemia by ER-dependent versus -independent mechanisms. We conducted studies to address the premise that estradiol may modulate NO and/or GAD neuron receptivity to NE. Using pharmacological and high-resolution microdissection tools in a characterized OVX, plus an estradiol replacement model in which plasma estradiol levels are reinstated within the physiological range [32,33,34], we investigated whether VMN ERα and/or ERβ modulate NE control of nitrergic and GABAergic neuron AR and transmitter marker protein expression in the female rat [35]. The research plan involved a pretreatment by intra-VMN administration of MPP, PHTPP, or vehicle, prior to the injection of NE into the same location. The VMN was sectioned into alternating thick versus thin frozen sections for respective harvesting of VMN tissue by Palkovits micropunch dissection technique for nNOS and GAD Western blot analysis versus laser-catapult microdissection of nNOS- or GAD_65/67_-immunoreactive (-ir) – neurons for nerve cell type-specific measurements of α_1_-AR, α_2_-AR, and β_1_-AR protein expression. The study outcomes provide unique proof that both ERs up-regulate basal VMN nNOS, but not GAD protein profiles, and that NE suppression or enhancement of nNOS and GAD expression was, respectively, ER-independent or -dependent. In both neuron types, VMN ERβ activity inhibited baseline α_1_- and/or α_2_-AR protein expression, but ERα and -β signaling was paradoxically crucial for the noradrenergic up-regulation of α_2_-AR. This work importantly documents distinctive ERα and ERβ actions on the metabolic transmitter and AR protein expression in VMN nitrergic versus GABAergic neurons. The interesting observation that ER actions varied in the presence versus absence of NE bolster the novel concept that NO and GABA neurons are substrates for critical estradiol and noradrenergic regulatory interaction.

**Estrogen regulation of VMN gluco-regulatory nerve cell AMPK reactivity to NE**. Using the same methodological approach for the selective procurement of VMN NO and GABA neurons and techniques for the administration of NE to the VMN [35], our laboratory examined the premise that one or both neuron populations express AMPK and that VMN ERs modulate the noradrenergic regulation of sensor protein expression and/or activation. Whereas both ERα and -β were observed to stimulate total AMPK protein expression in nitrergic neurons under control conditions, this protein profile was diminished in response to NE, an action that required ERβ signaling. With respect to NO nerve cell phospho-AMPK (pAMPK) levels, our studies show that baseline profiles are subject to an ERβ inhibitory tone, but that ERα signaling is stimulatory to pAMPK expression when NE is present. GABAergic nerve cell AMPK levels were observed to be suppressed by ERβ with or without NE stimulation; noradrenergic stimulation did not modify total AMPK protein expression. NE administration decreased pAMPK content in GABA cells by ER-independent mechanisms. These results importantly document AMPK expression in VMN nitrergic and GABAergic neurons, evidence that is consistent with cellular metabolic-sensory functionality. The outcomes also show that the NE regulation of the sensor activity state differs between these two cell types, e.g., the noradrenergic influence on total AMPK versus the pAMPK protein expression in NO versus GABA cells, respectively. Moreover, the results point to a noradrenergic-mediated switch in the balance (positive to negative) of the ERβ impact on NO AMPK protein profiles, which supports the existence of an estrogenic/noradrenergic signal interface; further studies are needed to elucidate the molecular mechanisms that underlie such communication. Taken together, these findings support the possibility that NE may link hindbrain and VMN AMPK-expressing neurons (Figure 1). The interpretation of research outcomes derived by the application of combinatory immunocytochemistry/laser-catapult microdissection/high-sensitivity Western blot techniques will be facilitated by corroborative results gained by alternative approaches. Further studies are needed to expand recent observations that estradiol governs VMN AMPK activity through direct control of the upstream regulatory kinase function and/or by its impact on energy metabolism to determine if such control underlies estradiol’s effects on these distinctive neuron populations [36].

**Noradrenergic regulation of VMN astrocyte glycogen metabolism**. Brain astrocytes store glycogen as a vital metabolic fuel reserve; this depot exhibits a dynamic turnover during normal brain activity and metabolic homeostasis, and is a critical source of lactate equivalents during heightened neural activity or brain glucose deficiency [37]. Circulating glucose, the primary energy source to the brain, is taken up into these glia where it is either incorporated into glycogen or converted into the oxidizable substrate fuel l-lactate for trafficking to neurons [38] by astrocyte (MCT1)- and the neuron (MCT2)-specific monocarboxylate transporter function [39]. Glycogen metabolism is controlled by opposing glycogen synthase (GS) and glycogen phosphorylase (GP) enzyme actions that catalyze glycogen synthesis or breakdown, respectively. Our findings that pharmacological suppression of VMN GP activity by 1,4-dideoxy-1,4-imino-d-arabinitol up-regulates the VMN nNOS expression in each sex (Alshamrani and Briski, personal communication) [40], and inhibits GAD profiles in females (Alshamrani and Briski, personal communication), imply that astrocyte glycogen-derived energy fuel streams affect neuro-metabolic stability in that structure, and that diminished astrocyte glycogen mass or turnover may be interpreted by VMN gluco-regulatory neurons as indicative of energy deficiency. NE has well documented regulatory effects on astrocyte glycogen metabolism in vitro [41,42]. We found that NE inhibits VMN GS protein expression, and promotes divergent adjustments in AMP-sensitive glycogen phosphorylase (GP)-brain type (up-regulated by NE) versus NE-sensitive GP-muscle (down-regulated by NE) variant profiles via ERα or -β activity, respectively [35]. These data uniquely demonstrate ER-dependent NE control of VMN GP variant expression. In light of proof that VMN astrocytes express these ERs and α_1_-AR, α_2_-AR, and β_1_-AR proteins, and that hypoglycemic patterns of AR variant expression are controlled by ERs [30], it is plausible that estradiol and NE signals may interact within these glia to direct physiological stimulus-specific control of glycogen mobilization (Figure 1).

**Noradrenergic regulation of VMN astrocyte AMPK activity is controlled by estradiol**. Recent studies indicate that the noradrenergic regulation of VMN energy sensor function is not exclusive to the nerve cell compartment. We carried out work involving the administration of the selective CA neurotoxin 6-hydroxydopamine (6-OHDA) into the caudal fourth ventricle of estradiol–versus non-hormone-replaced OVX female rats to address the premise that VMN astrocytes express AMPK, and that the reactivity of this sensor to hypoglycemia is estradiol- and/or hindbrain NE-dependent [43]. Individual VMN astrocytes identified in situ by immunolabeling for the glial marker fibrillary acidic protein were laser-microdissected for a Western blot analysis of AMPK and pAMPK protein expression. In these glia, baseline AMPK and pAMPK content was correspondingly higher or lower in estradiol- versus non-steroid-treated animals; interestingly, estradiol did not impact these astrocyte protein profiles in other hypothalamic metabolic structures. In the presence of estradiol, VMN astrocyte AMPK protein was decreased after singular exposure to either 6-OHDA or IIH. Importantly, hypoglycemia augmented pAMPK expression in these glia in vehicle-, but not 6-OHDA-pretreated estradiol-implanted animals. These outcomes provide novel proof of a hindbrain catecholamine-reliant activation of VMN astrocyte AMPK by hypoglycemia in the presence of estrogen. Additional studies corroborate these findings with proof that hypoglycemia stimulates VMN astrocyte AMPK and pAMPK profiles in female, but not male rats, and that these sex-dimorphic sensor activity responses are mediated by ERs [30]. Data reported by Ibrahim et al. [24] show that AMPK is involved in the NE regulation of VMN astrocyte expression of plasma membrane estrogen receptor GPER and β_1_-AR protein expression, as well as in GS and GP profiles. Collectively, our research implicates AMPK in the noradrenergic control of VMN astrocyte glycogen synthesis and metabolism. Distinguishing effects of NE on distinctive VMN astrocyte adrenergic and estrogen receptor variants support the possibility that AMPK regulation of glycogen metabolism in the presence of NE may be mediated, in part, by one or more receptors characterized in our work by sensitivity to this catecholamine.

Adrenergic receptor regulation of VMN gluco-regulatory nerve cell AMPK during acute versus recurring insulin-induced hypoglycemia (RIIH). RIIH can trigger hypoglycemia-associated autonomic failure in male type 1 diabetes patient, a pathophysiological mal-adaptation that manifests as diminished hypoglycemic awareness and defective glucose counter-regulation [44,45]. In vivo laboratory models for RIIH that replicate the insulin route of administration, injection frequency, and formulation duration of action in the clinical setting reveal a diminished nerve cell transcriptional activation in brain metabolic structures, an outcome that infers neurological desensitization to hypoglycemia [46,47]. While the possibility cannot be ruled out that multiple cellular, e.g., sensory, integrative, and motor components of the neural gluco-regulatory circuitry, may exhibit habituation to RIIH, available evidence points to the liable emergence of malfunctioning metabolic monitoring [48]. Our laboratory sought to investigate the hypothesis that NE controls VMN NO and GABA neuron AMPK activity during acute hypoglycemia through α_1_-AR signaling and that this receptor mediates acclimated energy sensor responses to RIIH [49]. Our research plan involved the pretreatment of male rats by *icv* delivery of the α_1_-AR reverse-agonist prazocin (PRZ) or vehicle ahead of induction of a single versus final episode (e.g., occurring on the fourth consecutive day of insulin administration) of hypoglycemia. VMN neurons identified by immunocytochemical labeling as nNOS- or GAD_65/67_-ir were procured by laser-catapult microdissection for Western blot analysis of nNOS or GAD profiles as well as AMPK, pAMPK, α_1_-AR, α_2_-AR, and β_1_-AR protein expression. The study results show that the hypoglycemic activation of VMN NO and GABA AMPK involves the α_1_-AR signal up-regulation of pAMPK profiles in nitrergic neurons or the down-regulation of the total AMPK protein in GABAergic nerve cells. The outcomes also reveal that antecedent hypoglycemia represses sensor activation in both cell groups during re-exposure to hypoglycemia. In nitrergic neurons, an acclimated sensor function may involve the loss of α_1_-AR stimulation of AMPK activity as PRZ did not modify sensor activation from control range during RIIH. Prior bouts of hypoglycemia may elicit, in the absence of α_1_-AR signaling to these cells, α_1_-AR-independent mechanisms that protect cellular energy balance and thus stabilize AMPK activity, during ensuing hypoglycemia. On the other hand, α_1_-AR-reliant and -non-reliant mechanisms may converge on AMPK, such that the latter remains operational during the pharmacological blockade of α_1_-AR. Indeed, AMPK is controlled by various endocrine and neurochemical signals alongside nutrient cues. Meanwhile, GABAergic neurons exhibit a switch from α_1_-AR stimulation to inhibition of AMPK activity during single versus serial exposure to hypoglycemia; additional work is needed to elucidate mechanisms that underlie this change in direction of α_1_-AR influence on AMPK activity state. Evidence that NO and GABA neurons express α_1_-AR supports the notion that PRZ treatment effects involve the direct action of this drug on each nerve cell type. It is noted that as this study involved *icv* PRZ delivery, the blockade of upstream α_1_-AR that controls afferent input may also contribute to the VMN neuron responses to this drug. We theorize that demonstrable adjustment from positive to negative α_1_-AR influence on GABA AMPK activity may involve downstream receptor and/or signal transduction pathway mechanisms. Yet, it is plausible that antecedent hypoglycemia may alter the increased α_1_-AR-mediated inhibitory neurochemical cues to VMN GABA AMPK. Our results indicate that VMN NO and GABA neurons exhibit common and dissimilar AR protein responses to hypoglycemia, as well as discrepant AR acclimation to RIIH. Both neuron populations showed α_1_-AR-driven augmentation of α_1_-AR protein during acute hypoglycemia, but this up-regulated response persisted in nitrergic, but not GABA cells during RIIH. Expression of α_2_-AR protein was refractory to acute hypoglycemia in both cell groups, but NO neurons showed an α_1_-AR-dependent increase in this profile during chronic hypoglycemia. Interestingly, NO (up-regulated) and GABA (down-regulated) nerve cell β_1_-AR expression diverged during both acute and recurring hypoglycemia. Collectively, these outcomes imply that antecedent hypoglycemia may augment noradrenergic input to nitrergic neurons as a consequence of increased α_2_-AR expression. Alternatively, GABA nerve cell habituation to RIIH may involve diminished α_1_-AR signaling. Nevertheless, the likelihood that post-receptor signal transduction pathways may acclimate to recurring hypoglycemia, independent of changes in receptor expression, cannot be disregarded.

**Estrogen regulation of hindbrain metabolic-sensory noradrenergic neuron energy metabolic adaptation to RIIH**. Evidence that estradiol-replaced OVX female rats exhibit no change in hypothalamic neuron transcriptional activation patterns and counter-regulatory hormone secretion between acute versus chronic exposure to hypoglycemia [26,27] supports the notion that estradiol can provide protection against neural acclimation to RIIH. Estradiol is a potent stimulus for energy metabolism in the brain, where it enhances the net substrate catabolism and oxidative respiration during bio-energetic instability due to a stroke [50]. Our research addressed the premise that the estradiol modulation of A2 NE signaling patterns between and during serial bouts of hypoglycemia correlates with hormonal neuro-protective effects on energy metabolic pathway functions [51]. Interestingly, the study outcomes show that an antecedent exposure to hypoglycemia elicited opposite adjustments in basal A2 neuron dopamine-β-hydroxylase protein expression in estradiol, versus oil-implanted OVX female rats and moreover, exacerbated these divergent adjustments during the re-induction of hypoglycemia. Estradiol was also observed to regulate RIIH effects on enzyme proteins involved in energy metabolism and fatty acid synthesis. As A2 cells from estradiol-implanted rats exhibit elevated baseline expression of the rate-limiting glycolytic enzyme phosphofructokinase in the aftermath of hypoglycemia, coincident with diminished C-V-alpha and ATP synthase-α protein profiles, we surmise that estradiol may stimulate glycolysis yet suppress mitochondrial aerobic respiration/energy production after recovery from this metabolic stress. During the fourth of four daily hypoglycemic episodes, estradiol was observed to enable alpha-ketoglutarate dehydrogenase up-regulation and to prevent the down-regulation of ATP synthase-α in OVX animals, results that imply that this hormone may beneficially alleviate energy state negativity in A2 cells during RIIH. This concept is bolstered by evidence that A2 AMPK activation is greater in oil- versus estradiol-implanted OVX rats during RIIH. Collectively, our results suggest that estradiol-mediated decrements in A2 noradrenergic signaling may reflect, in part, the energetic resilience of these cells to recurring hypoglycemia. Further research is warranted to determine if and how estradiol-controlled patterns of NE transmission during RIIH affect cellular metabolic stability and energy sensor functionality in A2 projection sites, including the VMN. There is also a need to ascertain whether estradiol acts directly on VMN and other hypothalamic nerve cell targets to stabilize or enhance energy metabolism in RIIH-exposed subjects. Furthermore, our studies bolster the need to consider how adaptive adjustments in between-hypoglycemia patterns of A2 neurotransmission may have physiological relevance for glucostatic as well as non-glucostatic neural functions controlled by this hindbrain cell group.

**Conclusions:** Growing proof of the physiological relevance of hindbrain AMPK to the neural control of energy and glucose homeostasis has generated interest in the characterization of signal types, e.g., nutrients, hormones, and neurotransmitters that converge to regulate the net activity of this sensor and neurochemical mechanisms of communication of activation status. Our studies demonstrate that hindbrain AMPK provides regulatory input to VMN AMPK, a concept that challenges the current view that the metabolic sensors in various neural structures provide autonomous input to downstream integrative and pre-motor elements of the neural gluco-regulatory circuitry. Departure from the norm of exclusive focus on hypothalamic, hindbrain, or portal vein sensors is expected to yield a unitary approach that addresses the novel prospect of sensor interaction and cooperation. This ‘inclusionist’ perspective represents a paradigm shift from the customary view of the hypothalamus as a self-contained, singular source of sensory indicators of cellular metabolic imbalance. We predict research outcomes which highlight that metabolic sensors assimilate unique combinations of metabolic stimuli, e.g., they are not inter-changeable, and that neural communication between sensors is a mechanism for disseminating distinctive signals and ultimately, a collective assessment of the body’s metabolic status. Hindbrain and hypothalamic metabolic-sensory neurons may plausibly operate as unique substrates for the as-yet incompletely understood protective actions of estradiol with respect to the defense of cellular and systemic energy homeostasis. Further insights concerning the molecular mechanisms of such hormone action, which will require the implementation of complimentary molecular, genetic, and electrophysiological approaches, will undoubtedly identify potential therapeutic targets for the enhancement of a positive energy balance during states of estrogen deficiency in women, and for the alleviation of maladaptive neural acclimation to recurring exposure to bio-energetic stressors.

## Figures and Tables

**Figure 1:**
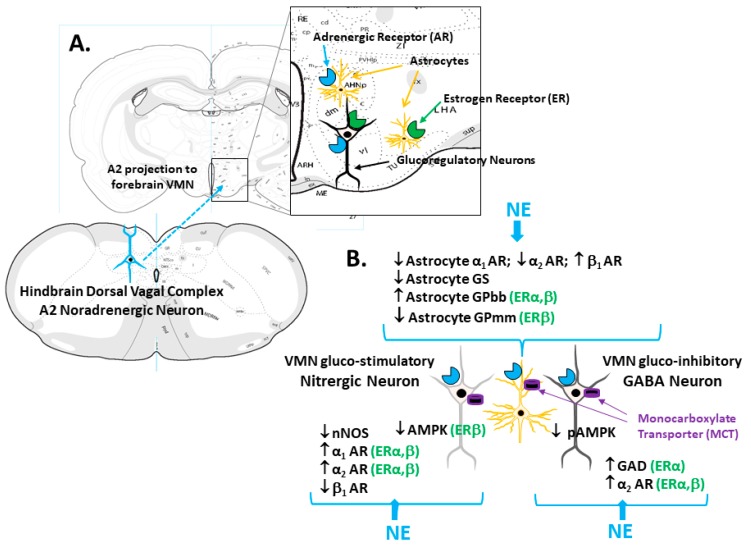
Model for Estrogen Receptor (ER) Modulation of Hindbrain A2 Noradrenergic Control of Ventromedial Hypothalamic Nucleus (VMN) Glucoregulatory Transmission. The enlarged rectangle in Panel A depicts ER and adrenergic receptor (AR) expression by VMN glucoregulatory neurons and astrocytes. Panel B illustrates ER-alpha (ERα) and/or ER-beta (ERβ)-dependent NE control of VMN astrocyte glycogen metabolic enzyme protein (glycogen phosphorylase-brain (GPbb) and -muscle (GPmm) type) expression. The same panel shows differential ER modulation of noradrenergic influence on gluco-stimulatory nitrergic (α_1_AR; α_2_AR; AMPK) versus gluco-inhibitory GABAergic (glutamate decarboxylase (GAD); α_2_AR) protein profiles. NE control of glucoregulatory signaling likely involves direct action on nitrergic and GABA neurons as well as regulation of astrocyte provision of glycogen-derived substrate fuel, e.g., lactate, by monocarboxylate transporter (MCT) function.

## Abbreviations

6-OHDA	6-hydroxydopamine
α_1_-AR	alpha_1_-adrenergic receptor
α_2_-AR	alpha_2_-adrenergic receptor
β_1_-AR	beta_1_-adrenergic receptor
AMPK	5′-AMP-activated protein kinase
CA	catecholamine
Cc	Compound C
ERα	estrogen receptor-alpha
ERβ	estrogen receptor-beta
GABA	γ-aminobutyric acid
GAD	glutamate decarboxylase_65/67_
GP	glycogen phosphorylase
GS	glycogen synthase
IIH	insulin-induced hypoglycemia
MBH	mediobasal hypothalamus
MPP	1,3-*Bis*(4-hydroxyphenyl)-4-methyl-5-[4-(2-piperidinylethoxy)phenol]-1*H*-pyrazole dihydrochloride
NE	norepinephrine
NO	nitric oxide
nNOS	neuronal nitric oxide synthase
OVX	ovariectomy
pAMKP	phospho-AMPK
PHTPP	4-[2-phenyl-5,7-*bis*(trifluoromethyl)pyrazolo[1,5-*a*]pyrimidin-3-yl]phenol
RIIH	recurrent insulin-induced hypoglycemia
VMN	ventromedial hypothalamic nucleus
